# Emerging 2D functional metal-organic framework materials

**DOI:** 10.1093/nsr/nwz159

**Published:** 2019-10-21

**Authors:** Bin Liang, Rui-Biao Lin, Banglin Chen

**Affiliations:** Department of Chemistry, University of Texas, San Antonio, USA

Two-dimensional metal-organic frame-works (2D MOFs) have been explored intensively in the fields of materials science, chemistry and nanotechnology over the past decade [1]. Their unique features such as the ultrathin thickness, more exposed accessible surface atoms and active sites have enabled 2D MOFs to exhibit superior applications for catalysis, sensing, gas separation and membrane devices [2,3].

Although top-down methods (such as chemical exfoliation, mechanical exfoliation and freeze–thaw exfoliation) or bottom-up methods (such as surfactant-assisted synthesis, interfacial synthesis, modulated synthesis and sonication synthesis) have been widely investigated for the fabrication of MOF nanosheets [4], it is still very challenging to prepare 2D MOFs systematically, rationally and straightforwardly. For example, the yield of the layered MOFs is quite low through the exfoliation method, while it is difficult to get high crystalline 2D MOFs through interfacial synthesis. Even for the well-developed surfactant-assisted method, the surface-active sites and pores of nanosheets of the resulting 2D MOFs might be partially blocked, which has limited their applications for catalysis or gas separation. It is thus very important to develop a facile and systematical methodology for the synthesis of high-quality 2D MOFs.

**Figure 1.**
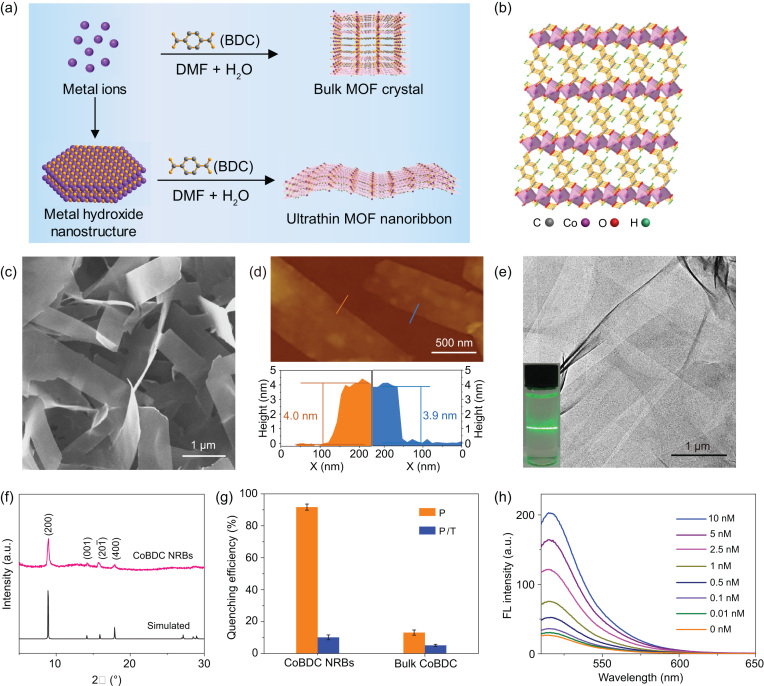
Design and characterization of MOF NRBs. (a) Comparison of the traditional approach to bulk MOF crystal and metal hydroxide nanostructure precursor approach to ultrathin MOF nanoribbons. (b) Crystal structure of CoBDC. (c) SEM images of the as-prepared CoBDC NRBs. (d) AFM images of CoBDC NRBs and the corresponding height profiles measured along the orange and blue lines, respectively. (e) TEM images of the as-prepared CoBDC NRBs. Inset in (e): the Tyndall effect of colloidal CoBDC NRBs in ethanol. (f) XRD pattern of the as-prepared CoBDC NRBs. (g) Fluorescence quenching efficiency of CoBDC NRBs and bulk CoBDC. (h) Fluorescence spectra of P (ssDNA probe) (10 nM) in the presence of different concentrations of T (target complementary DNA) with the addition of CoBDC NRBs. Reprinted from Wang *et al.* [5] with the permission of Oxford University Press.

In a recent work published in NSR by Wang *et al.*, ‘Ultrathin metal-organic framework nanoribbons’ [5], the resear-chers realized a general and efficient approach for fabricating novel ultrathin MOF materials and proved its excellent performance in DNA detection (Fig. 1). In this work, various kinds of ultrathin MOF nanoribbons (NRBs) such as NiBDC (BDC = 1,4-benzenedicar-boxylate), NiCoBDC, CoTCPP (TCPP = tetrakis(4-carboxyphenyl)porphyrin) and MIL-53(Al) NRBs have been successfully synthesized. It is worth pointing out that the precursors of metal hydroxide nanostructures were demonstrated to play a significant role in the formation of MOF NRBs. They can provide nucleation sites for the initial growth of the ultrathin MOF NRBs and achieve controlled release of the desired metal ions during the formation process of ultrathin MOF NRBs.

The as-prepared CoBDC NRBs were used for the application of DNA detection. With the single-stranded DNA (ssDNA) probes on their surface, the CoBDC NRB was successfully used as the sensing platform. The quantitative detection of the target DNA can be achieved by measuring the fluorescence intensity of the probe. For the fluorescence quenching testing, the results showed that the CoBDC NRBs exhibit better performance than the bulk CoBDC MOFs materials (Fig. 1g). Compared with the existing materials of DNA detecting, the CoBDC NRBs also exhibited very good sensitivity for the target DNA (Fig. 1g and h).

Overall, this work represents very important progress in MOF chemistry and 2D nanoscale materials through the facile and straightforward approach to preparing ultrathin 2D MOF materials. This pioneering work will significantly facilitate the development and implementation of 2D multifunctional MOF materials for diverse applications that will be beyond those developed on gas separation, catalysis, sensing and membrane devices in the future.

***Conflict of interest statement***. None declared.
